# UHPLC-HRMS and GC-MS Screening of a Selection of Synthetic Cannabinoids and Metabolites in Urine of Consumers

**DOI:** 10.3390/medicina56080408

**Published:** 2020-08-13

**Authors:** Manuela Pellegrini, Emilia Marchei, Esther Papaseit, Magí Farré, Simona Zaami

**Affiliations:** 1 National Centre on Addiction and Doping, Istituto Superiore di Sanità, V.Le Regina Elena 299, 00161 Rome, Italy; manuela.pellegrini@iss.it (M.P.); emilia.marchei@iss.it (E.M.); 2 Clinical Pharmacology Unit, Hospital Universitari Germans Trias i Pujol and Institut de Recerca Germans Trias i Pujol (HUGTiP-IGTP), 08916 Badalona, Spain; epapaseit.germanstrias@gencat.cat (E.P.); mfarre.germanstrias@gencat.cat (M.F.); 3 Department of Pharmacology, Therapeutics and Toxicology, Universitat Autònoma de Barcelona, 08193 Cerdanyola del Vallés, Spain; 4 Department of Anatomical, Histological, Forensic and Orthopedic Sciences, Sapienza University, 00161 Rome, Italy

**Keywords:** synthetic cannabinoids, urine, liquid chromatography, high-resolution mass spectrometry, gas chromatography-mass spectrometry

## Abstract

*Background and Objectives*: The use of synthetic cannabinoids has increased around the world. As a result, the implementation of accurate analysis in human biological matrices is relevant and fundamental. Two different analytical technologies, ultra-high-performance liquid chromatography-high-resolution mass spectrometry (UHPLC-HRMS) and high-sensitivity gas chromatography-mass spectrometry (GC-MS) were used for the determination of three synthetic cannabinoids JWH-122, JWH 210, UR-144 and their metabolites in urine of consumers. *Materials and Methods*: Sample preparation included an initial hydrolysis with β-glucuronidase and liquid-liquid extraction. The UHPLC-HRMS method included a Kinetex 2.6 u Biphenyl 100A (100 × 2.1 mm, 2.6 μm) (Phenomenex, Italy) column with a gradient mobile phase consisting of mobile phase A (ammonium formate 2mM in water, 0.1% formic acid) and mobile phase B (ammonium formate 2mM in methanol/acetonitrile 50:50 (v/v), 0.1% formic acid) and a full-scan data-dependent MS2 (ddMS2) mode was used (mass range 100–1000 m/z). The GC-MS method employed an ultra-Inert Intuvo GC column (HP-5MS UI, 30 m × 250 µm i.d, film thickness 0.25 µm; Agilent Technologies, Santa Clara, CA, USA) and electron-impact (EI) mass spectra were recorded in total ion monitoring mode (scan range 40–550 m/z). *Results:* Both methods have been successfully used for screening of parent synthetic cannabinoids and their metabolites in urine samples of consumers. *Conclusions:* The screening method applied JWH-122, JWH-210, UR-144 and their metabolites in urine of consumers can be applied to other compounds of the JWH family.

## 1. Introduction

Over the last few years, synthetic cannabinoids, also called synthetic cannabinoid receptor agonists, have been introduced on the illicit market to evade psychotropic drugs legislation and increase the onset and the duration of action of cannabis effects [1,2].

The rapid synthesis of such compounds and their rising popularity in illegal markets have become a challenge for clinical and forensic laboratories, since the development of analytical methods cannot keep up with the rapid change of chemical structures. Indeed, 14 chemical families of synthetic cannabinoid receptor agonists are presently recognized and not all are included in current banning laws [1,3]. Similar to the other new psychoactive substances, the analytical detection of synthetic cannabinoids presents several limitations, the most important being the unavailability of adequate reference standards for parent drugs and metabolites and the lack of analytical methods to readily detect these substances in cases of intoxication and fatalities [4]. Differently from cannabis products, synthetic cannabinoids can cause severe toxicity in active consumers and the offspring of pregnant or breastfeeding mothers [5,6], causing outbreaks of intoxication and fatalities [7,8]. Therefore, the analytical challenge involves not only the large range of different compounds and/or metabolites to identify, but also the variety of biological matrices to investigate including non-conventional matrices, which have gained major interest in recent years for information provided, detection window and minimal sample collection invasiveness [9].

A variety of rapid test kits have been developed and marketed over the past few decades, most of which are only intended for rapid and presumptive identification of traditional drugs of abuse. However, the specificity of these methods depends on the affinity and the cross-reactivity of the antibodies used for the parent drug, its analogues and its metabolites. Initial screening of synthetic cannabinoids and their metabolites in biological matrices is difficult since the few onsite or immunochemical tests available on the market are specific for single molecules and often only for parent drugs [10]. The rapid and continuous release of novel molecules and the need to detect not only parent drugs but also and especially drug metabolites require more specific and selective methodologies such as gas or liquid chromatography coupled to mass spectrometry or tandem mass spectrometry [11,12,13,14,15,16]. We hereby propose a screening method for urinalysis of synthetic cannabinoids and principal metabolites using a fast sample extraction and two different analytical techniques: ultra-high-performance liquid chromatography-high-resolution mass spectrometry (UHPLC-HRMS) and high-sensitivity gas chromatography-mass spectrometry (GC-MS). The developed methodology has been used for the rapid screening of three synthetic cannabinoids JWH-122, JWH-210 UR-144 and their respective metabolites JWH-122 N-(4-hydroxypentyl), JWH-122 N-(5-hydroxypentyl), JWH-210 N-(4-hydroxypentyl), JWH-210 N-(5-hydroxypentyl), UR-144 N-(4-hydroxypentyl) and UR-144 N-(5-hydroxypentyl) in urine of Spanish consumers. We focused on these particular substances due to their recent spread on the Spanish illegal market, as reported by the same consumers in web fora and to a Spanish Non Governmental Organization dealing with drug risk reduction.

## 2. Methodology

### 2.1. Sample Preparation

Urine samples collected between 29 March 2019 and 10 May 2019 were donated by synthetic cannabinoid consumers, who attended at a private club. Each participant self-administered a cigarette containing the synthetic cannabinoid they selected which was obtained from an unknown source, but analysed for content by a Drug Checking Service performed by a Spanish ONG (Energy Control). At the time of the study, none of these synthetic cannabinoids were illegal in Spain and personal use was allowed in private clubs for cannabis and synthetic cannabinoid users. Sample collection was authorized by the local Human Research Ethics Committee (CEI-HUGTiP ref. PI-18-267, Badalona, Spain). Samples were stored at −20 °C until analysis.

Analytes were retrieved by liquid-liquid extraction. Since it has been shown that synthetic cannabinoid hydroxymetabolites are mainly present as glucuronides in urine samples, urine hydrolysis was performed as reported in previous studies [12,14,15].

A volume of 2 mL urine was mixed with 200 μL β-glucuronidase (type H2 from *Helix Pomatia* (110,200 units/mL, Sigma-Aldrich, Milan, Italy) in 0.1 M phosphate buffer (pH 4). Hydrolysis of conjugates was achieved by incubation at 60 °C for 2 h.

After cooling, samples were extracted twice with 6 mL hexane/ethyl acetate (9:1). After centrifugation, the organic layer was divided in two aliquots of 3 mL each.

The first aliquot was evaporated to dryness at 40 °C under a nitrogen stream and derivatized with 25 μL Bis(trimethylsilyl)trifluoroacetamide (BSTFA) containing 1% trimethylchlorosilane (TMCS) at 70 °C for 30 min. A volume of 1 μL was injected into the GC-MS system.

The second aliquot was evaporated to dryness under a nitrogen stream and then dissolved in 50 μL mixture of mobile phase A (ammonium formate 2 mM in water, 0.1% formic acid) and B (ammonium formate 2 mM in methanol/acetonitrile 50/50, 0.1% formic acid) (50:50, v/v). A volume of 10 μL was injected into UHPLC-HRMS.

### 2.2. Gas Chromatography-Mass Spectrometry (GC-MS) Instrumentation

The GC-MS instrument consisted of an Intuvo 9000 GC System coupled with 5977 B MSD (Agilent Technologies, Palo Alto, CA, USA). The Ultra-Inert Intuvo GC column (HP-5MS UI, 30 m × 250 µm i.d, film thickness 0.25 µm; Agilent Technologies) was used for separation. The GC-MS conditions for the screening procedures were as follows: split-less injection mode; helium (purity 99%) carrier gas flow 1.2 mL/min; injection port, ion source, quadrupole, and transfer line temperatures were 260, 230, 150 and 320 °C, respectively; column temperature was 70 °C for 2 min and increased to 190° C at 30 °C/min and then increased to 290 °C at 5 °C/min for 10 min. Subsequently, the temperature was increased to 340 °C at 40 °C/min to eliminate impurities from the column. The electron-impact (EI) mass spectra were recorded in total ion monitoring mode (scan range 40–550 m/z).

Full scan data files were processed by Agilent MassHunter Workstation–Unknowns Analysis (Agilent Technologies). The mass spectra international libraries used for peak identification were NIST Research Library (National Institute of Standards and Technology) and SWGDRUG Library version 3.6 (Scientific Working Group for the Analysis of Seized DrugsWebsiteswgdrug.org).

### 2.3. Ultra-High-Performance Liquid Chromatography-High-Resolution Accurate Masses Spectrometry (UHPLC-HRMS) Instrumentation

The UHPLC/ESI Q-Orbitrap system consisted of an Ultimate 3000 LC pump and an Ultimate 3000 autosampler coupled with a Q Exactive Focus mass spectrometer equipped with a heated electrospray ionization (HESI) probe operating in positive ionization mode, and the system was controlled by Trace finder 4.0 software (ThermoFisher Scientific, Bremen, Germany).

Separation was performed on a Kinetex Biphenyl 100A (100 × 2.1 mm, 2.6 μm) (Phenomenex, Italy). The run time was 18 min with a gradient mobile phase composed of ammonium formate 2 mM in water with 0.1% formic acid (mobile phase A) and ammonium formate 2 mM in methanol/acetonitrile 50:50 (v/v) with 0.1% formic acid (mobile phase B) at a flow rate of 0.6 mL/min. Initial conditions were 20% B, held for 2 min, increased to 81.4% B within 9 min, increased to 100% B within 0.2 min, held for 4.3 min, returned to 20% B within 0.1min, and then held for 2.4 min. LC flow was directed to waste for the first 4.5 min and after 13.5 min. Autosampler and column oven temperatures were 4 °C and 40 °C, respectively.

MS parameters were as follows: ionization voltage was 3.0 kV; sheath gas and auxiliary gas were 35 and 15 arbitrary units, respectively; S-lens radio frequency RF level was 60; vaporizer temperature and capillary temperature were 320 °C. Nitrogen was used for spray stabilization, for collision induced dissociation experiments in the higher-energy collisional dissociation (HCD) cell and as the damping gas in the C-trap. The instrument was calibrated in positive and negative modes every week.

Data were acquired in full-scan data-dependent MS2 (ddMS2) mode with an inclusion list containing the exact masses of over 1400 compounds including parent compounds and their metabolites. Full scan data acquisition was conducted as follows: resolution of 70,000, micro-scans of 1, maximum injection time of 120 ms and a scan range of 100–1000 m/z.

The following settings for the dd-MS^2^ mode were used: resolution of 17,500, isolation window of 1.0 and HCD cell with stepped normalized collision energy of 17.5, 35.0, 52.5 V.

The MS and fragmentation data acquired were processed by Thermo Scientific TraceFinder software. This specific software performs a thorough interrogation of the database by making use of the built-in database and mass spectral library of over 1400 compounds, retention times, isotope pattern matching, and elemental composition determinations to identify drugs and metabolites.

### 2.4. Short Methods Validation

Although a complete methods validation was not carried out, since proposed methodologies were only intended for initial screening of urine samples, relevant validation parameters were determined following the most recent criteria for method development and validation in analytical toxicology [17,18]. Limits of Detection (LODs) and Limits of Quantification (LOQs) were estimated by analyzing a pool of blank urine samples with decreasing concentrations of the spiked analytes and thereafter calculating the signal to noise ratio. LOD was defined as the lowest concentration with good chromatography that yielded a signal-to-noise ratio higher than 3 and LOQ the lowest concentration with a signal-to-noise ratio higher than 10. Limits of detection and quantification, carry over, and selectivity were also calculated, as they are essential parameters for a screening methodology.

## 3. Results

A simple and selective screening analysis with simultaneous use of high-sensitivity GC-MS and UHPLC-HRMS was applied for the determination of synthetic cannabinoids JWH-122, JWH-210 UR-144 and their respective metabolites JWH-122 N-(4-hydroxypentyl), JWH-122 N-(5-hydroxypentyl), JWH-210 N-(4-hydroxypentyl), JWH-210 N-(5-hydroxypentyl), UR-144 N-(4-hydroxypentyl) and UR-144 N-(5-hydroxypentyl) in urine. Particularly, hydroxy metabolites have been chosen as the main metabolites of parent synthetic cannabinoids as reported in previous studies [13,14,15,16]. The procedure consisted of a two-hour hydrolysis step followed by an extraction lasting 15 min. Due to the unavailability of glucuronated metabolites, hydrolysis efficiency could not be established, but in agreement with previous studies, after hydrolysis hydroxymetabolite urinary concentration increased by 30% [14,15,16].

**Figure 1:**
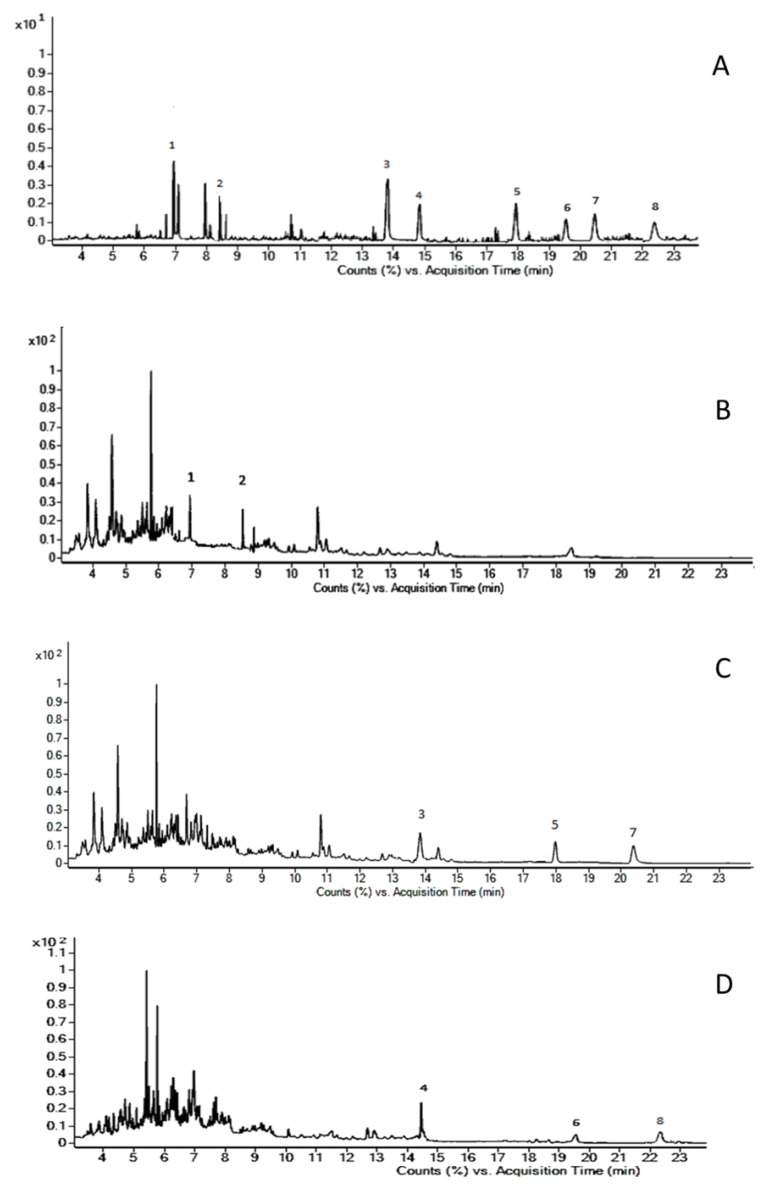
**Gas chromatography-mass spectrometry** (GC-MS) total ion chromatograms of: (**A**) Extract of 1 mL drug-free urine spiked with 0.3 ng of UR-144 (1), UR-144 N-(5-hydroxypentyl) (2), JWH-122 (3), JWH-210 (4), JWH-122 N-(4-hydroxypentyl) (5), JWH-210 N-(4-hydroxypentyl) (6), JWH-122 N-(5-hydroxypentyl) (7) and JWH-210 N-(5-hydroxypentyl) (8). (**B**) Urine sample positive to UR-144 (1) and UR-144 N-(5-hydroxypentyl) (2); (**C**) Urine sample positive to JWH-122 (3), JWH-122 N–(4-hydroxypentyl) (5) and JWH-122 N-(5-hydroxypentyl) (7); (**D**) Urine sample positive to JWH-210 (4) and JWH-210 N-(4-hydroxypentyl) (6) and JWH-210 N-(5-hydroxypentyl) (8).

**Figure 2:**
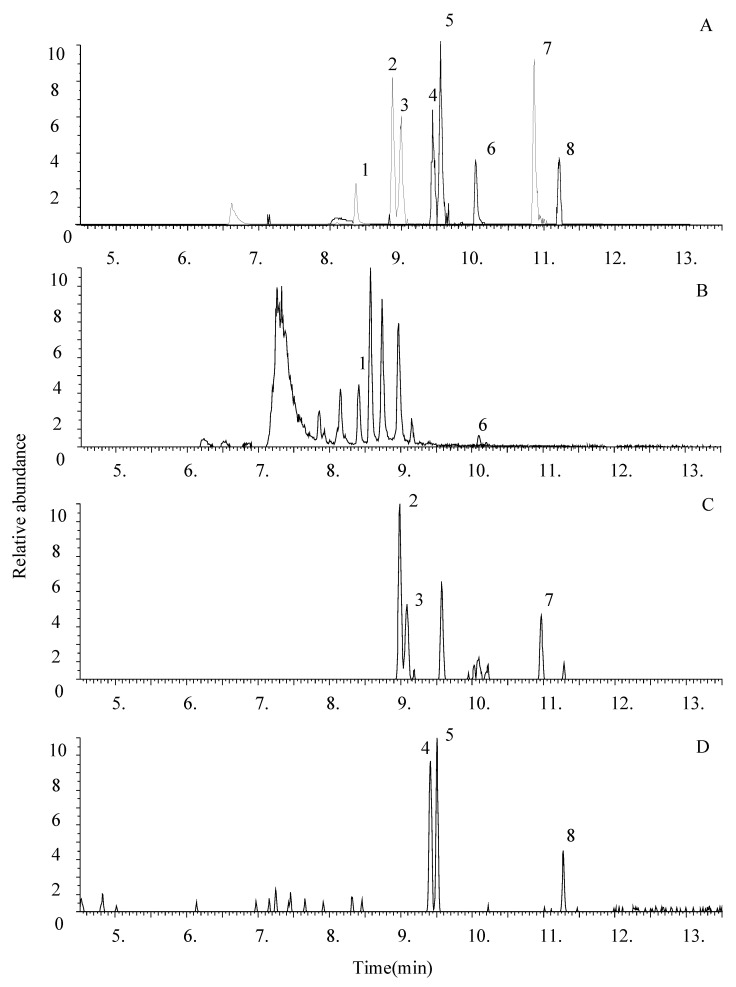
Representative range mass overlay ultra-high-performance liquid chromatography-high-resolution mass spectrometry (UHPLC-HRMS) chromatograms of urine. (**A**) Extract of 1mL urine spiked with 0.3 ng of UR-144 5N hydroxy pentyl (1), JWH-122 4N hydroxy pentyl (2), JWH-122 5N hydroxy pentyl (3), JWH-210 4N hydroxy pentyl (4), JWH-210 5N hydroxy pentyl (5), UR-144 (6), JWH-122 (7) and JWH-210 (8), (**B**) urine sample positive to UR-144 (6) and UR-144 5N hydroxy pentyl (1); (**C**) Urine sample positive to JWH 122 (7) and JWH-122 4N hydroxy pentyl (2), JWH-122 5N hydroxy pentyl (3); (**D**) urine sample positive to JWH 210 (8) and JWH-210 4N hydroxy pentyl (4), JWH-210 5N hydroxy pentyl (5).

After sample pretreatment, the first aliquot of extracted samples is immediately injected into the UHPLC-HRMS system, for a 20-min run. The second aliquot undergoes a 30-min derivatization followed by a 20-min GC/MS run. Extracted ion chromatograms from the extraction of 1-mL drug-free urine spiked with 100 ng UR-144, JWH-122, JWH-210 and their metabolites, and three positive urine samples screened with the two different instruments, are shown in Figure 1 and Figure 2.

Retention times monitored m/z ions, and limits of detection (LOD) and quantification (LOQ) of the analytes under investigations applying the two different methodologies are reported in Table 1.

Although a proper method validation was not carried out, essential parameters were evaluated. LOD and LOQ obtained for all the analytes under investigation fitted the purpose of the study. No additional peaks due to endogenous substances and carryover interfering with analytes were detected.

Furthermore, even if the total analysis time was not short, this methodology was not set up only to screen the compounds reported in this study, which were only an example. Indeed, preliminary experiments showed that the same methodology could be applied for all JWH families (e.g., JWH 018, JWH 073, JWH 200, JWH 250) tested as pure standards at the time of publication. In this concern it has to be said that two different analytical methodologies with different features were applied to screen the reported compounds. On one hand, UHPLC-HRMS provided a faster run time and it can be applied also in cases where pure standards of parent substances and metabolites under investigation are not available, since molecular ion exact mass measurement and an extended spectra library allowed a good recognition of several synthetic cannabinoids and principal metabolites. On the other, a last generation GC-MS assay provided same sensitivity (in terms of LOD and LOQ) and specificity of UHPLC-HRMS, showing that a more traditional, cheaper, simpler and widespread methodology can also be applied to screen these new psychoactive substances and metabolites in biological fluid.

This evidence is important in clinical and forensic cases involving intoxication or fatalities especially when the consumer is unaware of consumed substances due to surreptitious product substitution or adulteration [19,20,21,22].

## 4. Conclusions

The limited availability of screening tests for the detection of synthetic cannabinoids and/or metabolites in urine of consumers [9,14] prompted us to propose a screening method for urinalysis of JWH-122, JWH-210, UR-144 and their metabolites. The method can be applied to other compounds of the JWH family and successfully coupled UHPLC-HRMS and GC/MS assays.

## Figures and Tables

**Table 1:** Retention time (Rt), monitored ions (m/z), Limit of Detection (LOD), Limit of Quantification (LOQ) of non-derivatized and derivatized (TMS) analytes in GC/MS and UHPLC-HRMS.

Compound	GC/MS				UHPLC-HRMS			
	Rt (min)	Monitored m/z Ions	LOD ng/mL	LOQ ng/mL	Rt (min)	Monitored m/z Ions	LOD ng/mL	LOQ ng/mL
**UR-144**	7.1	144, 214, 296	0.1	0.3	10.1	55.0551, 125.0961, 244.0963, 312.2321	0.1	0.3
**UR-144 N-(5-hydroxypentyl) TMS**	8.7	302, 384, 399	0.1	0.3	8.2	55.0551, 125.0961, 230.1172, 328.2271	0.1	0.3
**JWH-122**	13.9	214, 284, 298	0.1	0.3	10.9	144.0443, 169.0646, 214.1223, 356.2008	0.1	0.3
**JWH-210**	14.8	144, 183, 214	0.1	0.3	11.2	155.0853, 183.0803, 214.1222, 370.2165	0.1	0.3
**JWH-122 N–(4-hydroxypentyl) TMS**	18.1	169, 284, 443	0.1	0.3	9.0	141.0697, 169.0646, 372.1958	0.1	0.3
**JWH-210 N-(4-hydroxypentyl) TMS**	19.6	183, 298, 457	0.1	0.3	9.4	144.044, 155.0853, 183.0803, 386.2114	0.1	0.3
**JWH-122 N–(5-hydroxypentyl) TMS**	20.6	169, 284, 443	0.1	0.3	9.0	141.0697, 169.0646, 372.1958	0.1	0.3
**JWH-210 N–(5-hydroxypentyl) TMS**	22.6	183, 298, 457	0.1	0.3	9.5	155.0853, 183.0803, 230.1171, 386.2114	0.1	0.3
